# Health Care Workers’ Reasons for Choosing Between Two Different COVID-19 Prophylaxis Trials in an Acute Pandemic Context: Single-Center Questionnaire Study

**DOI:** 10.2196/23441

**Published:** 2021-02-25

**Authors:** Alberto M Borobia, Irene García-García, Lucía Díaz-García, Amelia Rodríguez-Mariblanca, Lucía Martínez de Soto, Jaime Monserrat Villatoro, Enrique Seco Meseguer, Juan J González, Jesús Frías Iniesta, Elena Ramírez García, Jose Ramón Arribas, Antonio J Carcas-Sansuán

**Affiliations:** 1 Clinical Pharmacology Department, La Paz University Hospital (IdiPAZ) School of Medicine, Universidad Autónoma de Madrid Madrid Spain; 2 Internal Medicine Department, La Paz University Hospital (IdiPAZ) School of Medicine, Universidad Autónoma de Madrid Madrid Spain

**Keywords:** clinical trials, COVID-19, health care worker, motivation, personnel, pre-exposure, professional practice, prophylaxis, SARS-CoV-2, volunteers, web-based survey, workplace safety

## Abstract

**Background:**

In April 2020, two independent clinical trials to assess SARS-CoV-2 prophylaxis strategies among health care workers were initiated at our hospital: MeCOVID (melatonin vs placebo) and EPICOS (tenofovir disoproxil/emtricitabine vs hydroxychloroquine vs combination therapy vs placebo).

**Objective:**

This study aimed to evaluate the reasons why health care workers chose to participate in the MeCOVID and EPICOS trials, as well as why they chose one over the other.

**Methods:**

Both trials were offered to health care workers through an internal news bulletin. After an initial screening visit, all subjects were asked to respond to a web-based survey.

**Results:**

In the first month, 206 health care workers were screened and 160 were randomized. The survey participation was high at 73.3%. Health care workers cited “to contribute to scientific knowledge” (n=80, 53.0%), followed by “to avoid SARS-CoV-2 infection” (n=33, 21.9%) and “the interest to be tested for SARS-CoV-2” (n=28, 18.5%), as their primary reasons to participate in the trials. We observed significant differences in the expected personal benefits across physicians and nurses (*P*=.01). The vast majority of volunteers (n=202, 98.0%) selected the MeCOVID trial, their primary reason being their concern regarding adverse reactions to treatments in the EPICOS trial (n=102, 69.4%).

**Conclusions:**

Health care workers’ reasons to participate in prophylaxis trials in an acute pandemic context appear to be driven largely by their desire to contribute to science and to gain health benefits. Safety outweighed efficacy when choosing between the two clinical trials.

## Introduction

Therapeutic and phase I clinical studies have primarily focused on motivations for participating in clinical trials. However, limited information is available regarding subjects’ motivators to participate in prophylaxis trials in an acute pandemic context, with particular reference to COVID-19, especially in Western countries. In this setting, when several trials compete in recruiting participants from the same center, the participants’ reasons for choosing one trial over the other can be very relevant.

Currently, no pre-exposure prophylaxis therapy has been approved for COVID-19, but numerous clinical trials have been initiated in Europe and the United States, most of them assessing the potential of hydroxychloroquine as a prophylactic agent (eg, EudraCT-2020-001565-37, EudraCT-2020-001536-98, NCT04352946, NCT04354870, and NCT04328467) [1-5].

As of April 21, 2020, two independent clinical trials to assess COVID-19 prophylaxis strategies among health care workers have been initiated at the Clinical Trials Unit, La Paz University Hospital (Madrid, Spain): the MeCOVID trial (melatonin vs placebo; EudraCT-2020-001530-35) and the EPICOS trial (a four-arm clinical trial comparing the efficacy of tenofovir disoproxil/emtricitabine, hydroxychloroquine, combination therapy, and placebo; EudraCT-2020-001385-11).

This study aimed to evaluate the motivations among health care workers at our hospital to participate in COVID-19 prophylaxis trials and the reasons to select one of the two trials currently underway at our hospital.

## Methods

A choice between the two clinical trials was offered to our hospital personnel through the internal news bulletin. All potential participants received the information regarding both clinical trials and had the opportunity to have any questions answered before choosing between the two studies. Their choices were not influenced by the team of investigators.

Participants were screened to their chosen trial. In both clinical trials, they underwent COVID-19 screening using a serologic rapid test (Orient Gene, Orient Gene Biotech; or Wondfo, Guangzhou Wondfo Biotech). All screened subjects were asked to complete a web-based survey after their screening visit.

The questionnaire consisted of 9 questions on participant demographics, professional designation and work site, knowledge of COVID-19, motivation to participate, reasons to choose one trial over the other, and treatment expectations (Multimedia Appendix 1).

The survey was administered as a Google form. An invitation email containing a link to the questionnaire was distributed among all screened volunteers. No reminder emails were sent to nonresponders. Participants’ responses were anonymized and maintained confidential in accordance with Google’s privacy policy.

Descriptive statistics including variables with percentage values were determined. We performed the Shapiro–Wilk test to determine whether age was normally distributed in our study population; accordingly, we rejected our null hypothesis in the test for normality *(P*<.001) and found that age was normally distributed.

Statistical analyses were performed using R software (version 3.6.3, The R Foundation). We analyzed differences between age and position using the Mann–Whitney *U* test and between sex and position using the chi-square test. Furthermore, we performed the Fisher exact test to analyze differences in the responses of physicians and nurses.

## Results

In the first month of recruitment, 206 health care workers were screened and 160 were randomized (MeCOVID: n=156, 97.5% and EPICOS: n=4, 2.5%). Volunteers selected the trial in which they wanted to participate, the vast majority (n=202, 98.0%) having selected the MeCOVID trial. Table 1 shows the distribution of survey outcomes in the sampling frame. Furthermore, Table 2 shows the main data and the survey findings stratified by designation (physicians vs nurses vs others).

**Table 1:** Distribution of outcomes in the sampling frame (N=206).

Questionnaire response status among health care workers		Participants, n (%)
**Returned the questionnaire**		151 (73.3)
	Complete	142 (68.9)
	Partial or break-off with sufficient information^a^	9 (4.4)
**Did not return the questionnaire (nonresponders)**		55 (26.7)
	Logged in to survey but did not complete any items^b^	16 (7.8)
	Returned responses^c^	29 (14.1)
	Invitation returned undelivered^d^	10 (4.8)

^a^Registered for the survey and responded to almost all questions (maximum of 2 questions not answered).

^b^Registered for the survey but returned no responses.

^c^Remaining sample (participants remaining after excluding those who returned the questionnaires, those who logged in to survey but did not complete any items, and those for whom the invitation was returned undelivered.

^d^Email invitation was returned undelivered. Email delivery failed owing to the use of an incorrect, outdated, or out-of-space email address.

**Table 2:** Main survey findings stratified by participant designation (N=151).

Survey findings		Participant designation				*P* value^a^
		Physicians (n=64)	Nurses^b^ (n=79)	Others^c^ (n=8)		
**Responder characteristics**						
	Sex (males), n (%)	27 (42.2)	9 (11.4)	1 (12.5)	.001	
	Age (years), median (IQR)	41 (31-51)	37 (29-46)	46 (31-56)	.14	
**Knowledge of COVID-19, n (%)**						.001
	Expert	39 (60.9)	16 (20.2)	1 (12.5)		
	Basic	25 (39.1)	55 (69.6)	7 (87.5)		
	Some	0 (0)	7 (8.9)	0 (0)		
	No knowledge	0 (0)	1 (1.3)	0 (0)		
**Site of work, n (%)**						.01
	Emergency department	8 (12.5)	16 (20.3)	6 (75.0)		
	Intensive care units	4 (6.3)	18 (22.8)	1 (12.5)		
	Hospitalization wards	31 (48.4)	26 (32.9)	1 (12.5)		
	External offices/other	21 (32.8)	19 (24.0)	0 (0)		
**Main motivators, n (%)**						.03
	Scientific knowledge	31 (48.5)	42 (53.2)	7 (87.5)		
	To prevent SARS-CoV-2 infection	13 (20.3)	20 (25.3)	0 (0)		
	To have access to a SARS-CoV-2 rapid test	18 (28.1)	9 (11.4)	1 (12.5)		
	Other	2 (3.1)	8 (10.1)	0 (0)		
MeCOVID trial participants, n		62	77	8		
**Reason to participate in the MeCOVID trial (multiple answer question),** **n (%)**						<.001
	Adverse reactions to drugs in the EPICOS trial	53 (85.5)	49 (63.6)	0 (0)		
	Melatonin being a sleep aid	6 (9.7)	17 (22.1)	1 (12.5)		
	Melatonin having a high efficacy	2 (3.2)	17 (22.1)	6 (75.0)		
	Contraindications regarding EPICOS drugs	1 (1.6)	4 (5.2)	0 (0)		
	Not reported	4 (6.5)	1 (1.3)	1 (12.5)		
**Expectations regarding the MeCOVID trial,** **n (%)**						.13
	Drug efficacy and safety	26 (42.0)	50 (64.9)	8 (100)		
	Drug efficacy with adverse reactions	2 (3.2)	14 (18.2)	0 (0)		
	No drug efficacy and no adverse reactions	31 (50.0)	13 (16.9)	0 (0)		
	No drug efficacy with adverse reactions	1 (1.6)	0 (0)	0 (0)		
	Not reported	2 (3.2)	0 (0)	0 (0)		

^a^*P* values refer to differences between the responses of physicians and nurses. “Others” have been excluded from the analysis owing to their small sample size.

^b^“Nurses” includes nurse practitioners (n=54) and nursing assistants (n=25).

^c^“Others” includes laboratory technicians (n=2) and ancillary personnel (n=6).

In total, 56 (37.1%) participants reported having expert knowledge of COVID-19, of whom 39 (69.6%) were physicians, 16 (28.6%) were nurses, and 1 (1.8%) had another designation. The main motivation for many to participate in the trials was “to contribute to scientific knowledge” (n=80, 53.0%), followed by “to avoid SARS-CoV-2 infection” (n=33, 21.9%) and “the interest to be tested for SARS-CoV-2 by a serologic rapid test” (n=28, 18.5%) (Multimedia Appendix 2).

Regarding the expected personal benefits, we observed significant differences (*P*=.01) in the responses of physicians and nurses: the main expected benefit among physicians (n=18, 54.6%) was to gain access to SARS-CoV-2 rapid tests, while that among nurses (n=20, 62.5%) was to prevent SARS-CoV-2 infection (Multimedia Appendix 3).

Among the 147 subjects in the MeCOVID trial, the main motivation to participate was “the fear to present any toxicity related to EPICOS treatments” (n=102, 69.4%), while that among participants in the EPICOS trial was “the belief that hydroxychloroquine and tenofovir/emtricitabine might be more effective than melatonin to prevent SARS-CoV-2 infection” (n=2, 50%).

Again, we observed significant differences in the responses of physicians and nurses with respect to their reasons for selecting the MeCOVID trial. In total, 53 (85.5%) physicians and 49 (63.6%) nurses reported choosing the MeCOVID trial owing to adverse reactions to drugs used in the EPICOS trial. Conversely, 17 (22.1%) nurses and only 2 (3.2%) physicians preferred the MeCOVID trial to the EPICOS trial owing to the higher efficacy of melatonin, and 17 (22.1%) nurses and only 6 (9.7%) physicians reported the use of melatonin as a sleep aid (*P*<.001).

We had expected most participants (n=84, 57.1%) to favor the MeCOVID trial primarily because melatonin would be efficacious and safe, with 44 (29.9%) participants believing that although melatonin would not be efficacious, it would at least not lead to adverse reactions. Most participants (n=3, 75.0%) favoring the EPICOS trial expected the treatment to not only be efficacious but also to lead to some adverse reactions.

## Discussion

### Principal Findings

Prevention of SARS-CoV-2 transmission among health care workers is key to managing COVID-19 outbreaks. Although the precise number of COVID-19–infected health care workers is lacking, and studies have reported this infection rate to be 20% in Spain, approximately 10% in Italy and the United States, 6% in the Netherlands, and 3.8% in China [6]. Drug prophylaxis is one of the measures proposed to prevent SARS-CoV-2 infection among health care workers, and it is a priority to obtain strong evidence for this intervention.

This study evaluated the motivations among health care workers at our hospital to participate in 1 of 2 COVID-19 prophylaxis trials—MeCOVID and EPICOS—and their reasons to choose one trial over the other, given the different characteristics of these trials. Survey participation was high (n=151, 73.3%), with a response rate of approximately 70%. In our opinion, the reason for this is the use of a web-based survey, which was easy to respond to (taking only approximately 5 minutes to complete).

Empirical studies have reported that altruism and self-interest are the two primary motivations for participants to enroll in unpaid clinical trials. This holds good in various clinical situations, including participation in prophylaxis and vaccines trials, and in trials conducted at different geographical locations [7-10].

To our knowledge, this study is the first of its kind to be conducted in an acute pandemic context. Herein, the motivations of health care workers, who are aware of the severity and complications of COVID-19, to participate in the trials were similar to those reported in other clinical situations; that is, to contribute to science (n=80, 53%) and to gain personal benefits (n=71, 47.0%), and no significant differences were observed in the responses of physicians and nurses. However, the expected personal benefits were significantly different between physicians and nurses, with physicians being more interested in gaining access to SARS-CoV-2 rapid tests and nurses expecting to prevent SARS-CoV-2 infection. Such a combination of motivators has also been reported among subjects participating in HIV prophylaxis trials and Ebola vaccine trials [11-14].

A major reason for participants to choose one trial over the other in this study was the fear of adverse reactions to drugs used in the EPICOS trial; hence, two-thirds of the volunteers opted to participate in the MeCOVID trial. Our volunteers seemed to participate in the MeCOVID trial because they perceived it as less risky (they opted for this trial despite one-third volunteers not expecting any treatment efficacy). Furthermore, the reasons to participate in the MeCOVID trial were significantly different between physicians and nurses, with physicians being more concerned about adverse drug reactions.

### Strengths and Limitations

Although our study has a large sample and a high survey participation rate, it has some limitations of note. There are some biases in our study: a selection bias owing to the single-center study design and a low participation rate in the EPICOS trial (n=4, 2.5%). However, one of our study objectives was to evaluate motivations of health care workers to participate in COVID-19 prophylaxis trials independent of the trial selected. A large number of physicians and nurses participated in our survey, which enabled us to compare the responses between them. Another study limitation is that we could not precisely measure the reception of the survey via email. To be conservative and to estimate the survey participation rate, we have included the number of emailed individuals in the denominator.

### Conclusions

Our results show that health care workers’ motivations to participate in prophylaxis trials in an acute pandemic context appear to be driven mostly by their desire to contribute to science and obtain some health benefits. When the opportunity to participate in various trials is offered, safety markedly outweighed efficacy among the participants. Most participants opted to participate in one study over the other for safety reasons, having selected the trial they perceived as less risky, even if they consider it less efficacious. 

## Appendix

Multimedia Appendix 1 Survey for volunteers participating in clinical trials for COVID-19 porphylaxis.

Multimedia Appendix 2 Main motivations to participate in the trials classified as to contribute to science and personal benefits, subgrouped by physician vs nurses.

Multimedia Appendix 3 Personal benefits to participate in the trials subgrouped by physician vs nurses.
