# Supplementary material for: Health Care Workers’ Reasons for Choosing Between Two Different COVID-19 Prophylaxis Trials in an Acute Pandemic Context: Single-Center Questionnaire Study
Source: J Med Internet Res. 2021 Feb 25;23(2):e23441. doi: 10.2196/23441 (PMC7909307; doi:10.2196/23441)
Supplement: Multimedia Appendix 1 [file jmir_v23i2e23441_app1.pdf]

HEALTHCARE WORKERS' REASONS FOR CHOOSING BETWEEN TWO DIFFERENT COVID-19 PROPHYLAXIS TRIALS IN AN ACUTE PANDEMIC CONTEXT

Multimedia Appendix 1

1- Survey (Original spanish versión)

2- English translation

Encuesta para voluntarios participantes en estudio de profilaxis para COVID19

HOSPITAL UNIVERSITARIO LA PAZ - UNIDAD CENTRAL DE INVESTIGACIÓN CLÍNICA Y ENSAYOS CLÍNICO (UCICEC)

Estimado voluntario, el equipo investigador de los ensayos clínicos de profilaxis para infección por SARS-Cov-2 (EPICOS y MECOVID) estamos realizando esta encuesta anónima para conocer las razones que os han llevado participar en uno de estos dos ensayos, así como las expectativas del tratamiento.
Os agradecemos vuestra participación en esta encuesta.
Atentamente,

Equipo Investigador

1. ¿En qué estudio estás participando?

- Ensayo Clínico EPICOS
- Ensayo Clínico MECOVID (melatonina)

2. Sexo

- Mujer
- Hombre

3. Edad

____

4. Estamento

- Facultativo
- Enfermería
- Auxiliar de enfermería
- Auxiliar administrativo
- Técnico de Laboratorio
- Técnico de Radiología
- Celador
- Otros

5. Localización

- Urgencias
- Planta de Hospitalización
- Planta de Críticos (UCI/Anestesia)
- Consultas Externas
- Otros

6. Grado de conocimientos sobre la enfermedad

- Me considero un experto en COVID19
- Tengo altos conocimientos de la enfermedad
- Tengo conocimientos básicos sobre la enfermedad
- Tengo algún conocimiento sobre la enfermedad
- No tengo ningún conocimiento sobre la enfermedad

7. ¿Cuál ha sido su principal motivación para participar en alguno de estos dos ensayos clínicos?

- Por la ciencia, el conocimiento y el bien común
- Para que me hicieran un test rápido de serología
- Para prevenir la infección por SARS-Cov-2
- Otros

Si Otros, indicar la razón principal

____
____
____
____
____

Razones para la elección de uno de los dos ensayos clínicos

A usted se le ha ofrecido participar en dos ensayos clínicos de profilaxis, y ha elegido uno de ellos. En este apartado se le pregunta sobre las razones principales de su elección

8. SOLO PACIENTES INCLUIDOS EN MECOVID

Cuales son las razones principales por la que ha elegido usted participar en MECOVID (melatonina)?

- [ ] Porque tenía contraindicación para el ensayo clínico ÉPICOS
- [ ] Para tomar una pastilla que me ayudara a dormir
- [ ] Por el riesgo y/o miedo a tener una reacción adversa con Truvada
- [ ] Por el riesgo y/o miedo a tener una reacción adversa con Hidroxicloroquina
- [ ] Porque considero que melatoinina puede ser más eficaz que Truvada y/o Hidroxicloroquina

8. SOLO PACIENTES INCLUIDOS EN EPICOS

Cuales son las razones principales por la que ha elegido usted participar en ÉPICOS (truvada y/o hidroxicloroquina)

- [ ] Porque tenía contraindicación para el ensayo clínico MECOVID
- [ ] Por el riesgo y/o miedo a tener una reacción adversa con Melatonina
- [ ] Porque considero que Truvada y/o Hidroxicloroquina puede ser más eficaz que melatonina

Expectativas sobre el tratamiento

9. Con relación a sus expectativas sobre el tratamiento que se evalúa en el ensayo en el que decidido participar, indique la opción que más se aproxima:

- ( ) Creo que el tratamiento va a ser eficaz previniendo la infección por SARS-Cov-2, además de seguro
- ( ) Creo que el tratamiento va a ser eficaz previniendo la infección por SARS-Cov-2, pero que pued[illegible] tener alguna reacción adversa
- ( ) Creo que el tratamiento NO va a ser eficaz, pero NO voy a tener ninguna reacción adversa
- ( ) Creo que el tratamiento NO va a ser eficaz, y además voy a tener alguna reacción adversa

ENGLISH TRANSLATION

SURVEY FOR VOLUNTEERS PARTICIPATING IN CLINICAL TRIALS FOR COVID-19 PROPHYLAXIS

LA PAZ UNIVERSITY HOSPITAL – CENTRAL RESEARCH AND CLINICAL TRIAL UNIT (UCICEC)

Deer volunteer, the research team conducting the clinical trials for SARS-Cov-2 infection prophylaxis (EPICOS y MECOVID) are performing this anonymous survey to get to know the reason why you chose to participate in one of the two clinical trials offered, along with your expectations about the treatment proposed.
Thank you for participating in this survey.
Kind regards,
Research team.

1. ¿In which study are you participating?

- o EPICOS Clinica Trial.
- o MeCOVID Clinica Trial (melatonin).

2. Gender

- o Female.
- o Male.

3. Age:____________

4. Healthcare Job Title

- o Physician.
- o Nurse Practitioner.
- o Nursing assistant.
- o Administrative Assistant.
- o Laboratory technicians.
- o Radiologic Technician.
- o Ancillary
- o Other: ___________

5. **Working location**

- o Emergency room.
- o Hospitalization ward.
- o Intensive care.
- o External Offices
- o Other:____________

6. **Insight into COVID-19.**

- o I consider myself an expert on COVID19.
- o I have a high knowledge of COVID-19.
- o I have basic knowledge of COVID-19.
- o I have some knowledge of COVID-19.
- o I have no knowledge of COVID-19.

7. **¿What was the main motive for you to participate in any of the two clinical trials?**

- o To contribute to scientific knowledge and the common good.
- o The interest to be tested for SARS-CoV-2 by a rapid test.
- o To prevent SARS-Cov-2 infection.
- o Other:_______________________

Reasons to choose one trial over the other.

You have been offered to participate in two prophylaxis clinical trials and you have decided to participate in one of them. The following questions inquire about the reason for your choice.

ONLY PERSONNEL PATICIPATING IN MeCOVID.

8. **¿Which are the main reasons why you chose to participate in the MeCOVID (melatonin) trial?**

- o I had contraindications to participate in the EPICOS clinical trial.
- o To receive and take pills that would help me to sleep.
- o Risks and/or fear to present any adverse reaction to Truvada.
- o Risks and/or fear to present any adverse reaction to Hydroxychloroquine
- o I consider melatonin might be more effective than Truvada and/or Hydroxychloroquine.

ONLY PERSONNEL PATICIPATING IN EPICOS

8. ¿Which are the main reasons why you chose to participate in the EPICOS (Tenofovir disoproxil/Emtricitabine and/or Hydroxychloroquine) **trial?**

- I had contraindications to participate in the MECOVID clinical trial.
- Risks and/or fear to present any adverse reaction to melatonin.
- I consider Truvada and/or Hydroxychloroquine might be more effective than melatonin.

Expectations about treatment

9. Choose the most suitable option between the following related to your expectations regarding the study treatment.

- I believe the treatment is going to be efficacious for preventing SARS-Cov-2 infection, and I also think is going to be safe.
- I believe the treatment is going to be efficacious for preventing SARS-Cov-2 infection, but I think it could cause me some adverse reaction.
- I believe the treatment is NOT going to be efficacious for preventing SARS-Cov-2 infection, but I think it is NOT going to cause me any adverse reaction.
- I believe the treatment is NOT going to be efficacious for preventing SARS-Cov-2 infection, and I think it is going to cause me some adverse reaction.
