# Supplementary material for: Health Care Workers’ Reasons for Choosing Between Two Different COVID-19 Prophylaxis Trials in an Acute Pandemic Context: Single-Center Questionnaire Study
Source: J Med Internet Res. 2021 Feb 25;23(2):e23441. doi: 10.2196/23441 (PMC7909307; doi:10.2196/23441)
Supplement: Multimedia Appendix 2 [file jmir_v23i2e23441_app2.doc]

## Multimedia Appendix 2

The "Other" option allowed survey participants to answer the question with information that was not listed as an option. Taking into account their responses, we classified motivators in physicians and nurses as “to contribute to science”, “personal benefits” or “both” (see table S1).

Personal benefits to participate in the trials subgrouped by physician vs nurses were also sub-classified as: “To prevent SARS-Cov-2 infection”, “To have access to a SARS-CoV-2 rapid test”, “Sleep aid” and “Other” (see table S2 in Multimedia Appendix 3).

This is the information enter by survey participants as **“other motivators to participate in the trial”:**

**Physicians (n=2)**

1. “The rejuvenating effect.”

- Classified as “personal benefits” in table S1.
- Sub-classified as “others” in table S2 in Multimedia Appendix 3.

1. “I had planned to take melatonin and if I participate in the trial I am also tested for SARS-CoV-2 (I would not have done it just to be tested for SARS-CoV-2).”

- Classified as “personal benefits” in table S1.
- Sub-classified as “Sleep aid” in table S2 in Multimedia Appendix 3. (The participant had planned starting taking melatonin which indication is insomnia).

**Nurses (n=10)**

1. “I was asked by a college to participate.”

- Classified as “personal benefits” in table S1.
- Sub-classified as “others” in table S2 in Multimedia Appendix 3.

1. “A combination of the three motivators.”

- Classified as “both” in table S1.
- Not included in table S2 in Multimedia Appendix 3.

1. “My parents are old, and I am afraid for them. I have been working in the emergency room since the beginning of the pandemic and have seen a lot of dramatic situations.”

- This answer has been classified as “to prevent SARS-Cov-2 infection” for general data analysis.
- Classified as “personal benefits” in table S1.
- Sub-classified as “to prevent SARS-Cov-2 infection” in table S2 in Multimedia Appendix 3.

1. “I sleep poorly and I have heard melatonin could help me.”

- Reclassified as “personal benefits” in table S1.
- Sub-classified as “Sleep aid” in table S2 in Multimedia Appendix 3.

1. “I work in the emergency room; I have lived unpleasant and very hard experiences. My parents are old, there is no treatment and I am afraid they might get infected.”

- This answer has been classified as “to prevent SARS-Cov-2 infection” for data analysis.
- Reclassified as “personal benefits” in table S1.
- Classified as “to prevent SARS-Cov-2 infection” in table S2 in Multimedia Appendix 3.

1. “I want to sleep better.”

- Reclassified as “personal benefits” in table S1.
- Sub-classified as “Sleep aid” in table S2 in Multimedia Appendix 3.

1. “A combination of the two motivators: be tested for SARS-CO-2 and help to develop a possible prophylactic treatment for this virus.”

- Reclassified as “Both” in table S1.
- Sub-classified as “others” in table S2 in Multimedia Appendix 3.

1. Help to fight this virus and have some direct or indirect personal benefit

- Reclassified as “Both” in table S1.
- Not included in table S2 in Multimedia Appendix 3.

1. “Get to know how melatonin can have some benefit against COVID.”

- Reclassified as “Contribute to Scientific knowledge” in table S1.
- Not included in table S2 in Multimedia Appendix 3.

1. No answer was provided.

**Table S1**. Main motivations to participate in the trials classified as to contribute to science and personal benefits, subgrouped by physician vs nurses.

| **Main motivators, n(%)** | **Physicians**  **(n =64)** | **Nurses ^a^**  **(n =79)** | ***P*** |
| --- | --- | --- | --- |
| Contribute to Scientific knowledge | 31 (48.4) | 43 (54.4) | .24 |
| Personal benefits ^b^ | 33 (51.6) | 32 (40.5) |
| Both | 0 | 3 (3.8) |
| NR | 0 | 1 (1.3) |

^a^ “Nurses” includes nurse practitioners and nursing assistants.

^b^ This category includes: (1) “to prevent SARS-Cov-2 infection”, (2) “To have access to a SARS-CoV-2 rapid test” and (3) some of the “other” answers considered personal benefits.
