# Supplementary material for: Health Care Workers’ Reasons for Choosing Between Two Different COVID-19 Prophylaxis Trials in an Acute Pandemic Context: Single-Center Questionnaire Study
Source: J Med Internet Res. 2021 Feb 25;23(2):e23441. doi: 10.2196/23441 (PMC7909307; doi:10.2196/23441)
Supplement: Multimedia Appendix 3 [file jmir_v23i2e23441_app3.doc]

## Multimedia Appendix 3

Table S2. Personal benefits to participate in the trials subgrouped by physician vs nurses.

| **Personal benefit, n(%)** | **Physicians**  **(n =33)** | **Nurses ^a^**  **(n =32)** | ***P*** |
| --- | --- | --- | --- |
| To prevent SARS-Cov-2 infection | 13 (39.4%) | 20 (62.5%) | .01 |
| To have access to a SARS-CoV-2 rapid test | 18 (54.6%) | 9 (28.1%) |
| Sleep aid | 1 (3.0%) | 2 (6.3%) |
| Other | 1 (3.0%) | 1 (3.1%) |

^a^ “Nurses” includes nurse practitioners and nursing assistants.
